# Cutting Cycles of Conditional Preference Networks with Feedback Set Approach

**DOI:** 10.1155/2018/2082875

**Published:** 2018-06-28

**Authors:** Zhaowei Liu, Ke Li, Xinxin He

**Affiliations:** ^1^Shandong University, Jinan, Shandong Province, China; ^2^Yantai University, Yantai, Shandong Province, China

## Abstract

As a tool of qualitative representation, conditional preference network (CP-net) has recently become a hot research topic in the field of artificial intelligence. The semantics of CP-nets does not restrict the generation of cycles, but the existence of the cycles would affect the property of CP-nets such as satisfaction and consistency. This paper attempts to use the feedback set problem theory including feedback vertex set (FVS) and feedback arc set (FAS) to cut cycles in CP-nets. Because of great time complexity of the problem in general, this paper defines a class of the parent vertices in a ring CP-nets firstly and then gives corresponding algorithm, respectively, based on FVS and FAS. Finally, the experiment shows that the running time and the expressive ability of the two methods are compared.

## 1. Introduction

The evil of graph exists in cycles [1–3]. Several famous problems in computer science just like satisfiability, knapsack, and graph three-colorability problem are all related to cycles. With the awkward cycles, the above-mentioned questions are difficult to deal with.

Due to the importance of the problem, it has been extensively studied, although the problem was proven to be NP-complete for general graphs. Moreover, many graph problems are polynomially solvable if restricted to instances of acyclicity or even low cyclicity.

Generally, deleting cycles is considered as feedback set problem applied in many fields, such as circuit testing and deadlock resolution. Analyzing manufacturing processes and computational biology is used to delete cycles. Some different exact and approximate algorithms have been proposed incipiently based on Branch-Prune and linear programming. Measure-and-Conquer techniques and local search approaches have also been employed as usual method.

Feedback set [4, 5] includes feedback vertex set (FVS) and feedback arc set (FAS) or feedback edge set problems, which are classical NP problems. For different situations which can be undirected or directed graph, equal or unequal weighted graph, proper approaches have been proposed, but there is no uniform method in all cases.

A conditional preference network [1], abbreviated as a CP-net, is a simple and intuitive tool of graph model [6], which can represent preferences of agent, so do learning and aggregation and suits for describing qualitative multiattribute decision-making preference with dependencies. It can be converted into a weighted directed graph under usual conditions. Since it is a graph model, there always exist cycles. That will produce an effect on consistency of CP-nets where one single decision value does not appear more than one time in an arbitrary order sequence or satisfiability where there exists some preference dominant ranking for each decision value in the decision space. Moreover, the above-mentioned algorithms of FVS and FAS are not applicable because CP-nets are not general graph model [6, 7].

For cutting the cycles of CP-nets, two methods are presented based on feedback vertex set (FVS) and feedback arc set (FAS). The following are the main contributions of this paper.

(1) As a FAS problem, based on context of attribute priority, parent of vertex with cycles is defined with formalization firstly. Arcs (edges) of CP-nets are deleted by the relation; then an algorithm is presented [8, 9].

(2) As a FVS problem, based on the context of attribute relation, concept of relation reduction is given. Vertices of CP-nets are deleted by the through relation reduction; then another algorithm is proposed [10].

The rest of the paper is constructed as follows. In Section 2, some related works are presented. In Section 3, we present the main and basic definitions used throughout the paper. In Sections 4 and 5, FAS and FVS are proposed to deal with cycle of CP-nets, respectively.  Section 6 presents the results of experiments. Finally, Section 7 summarizes the work and present studies in the future work.

## 2. Related Work

In [11], Brafman and Domshlak tackle the complexity of determining whether one outcome is preferred to another outcome (dominance testing) which is known for tree-structured networks only; moreover, little is known about the consistency of cyclic CP-nets. In this paper they show how the complexity of dominance testing depends on the structure of the CP-net. In particular they provide a new polynomial time algorithm for polytrees. In addition, they show a class of cyclic CP-nets that is never consistent, while showing other classes on which consistency can be tested for efficiently.

The cyclic networks part of Domshlak in [1] proves that the consistency of cyclic CP-nets is not guaranteed and depends on the actual nature of the CPTs. This article holds that cyclic CP-nets usefulness requires further analysis. One can argue that it is possible to cluster the variables to preserve acyclicity. And it shows that further investigation of cyclic CP-nets, as well as a characterization of the different classes of utility functions that can be represented by cyclic and acyclic networks, remains of interest.

In [12], Liu et al. utilize treewidth which can decrease the solving complexity to solve some reasoning tasks on induced graphs, such as the dominance queries on the CP-nets in the future. And they present an efficient algorithm for computing the treewidth of induced graphs of CP-nets. It is revealed that by experiment the treewidth of induced graphs of CP-nets is much smaller with regard to the number of vertices.

## 3. Preliminaries

In this section, some basic concepts of CP-nets are presented.

**Definition 1 (CP-net).**

A conditional preference network (CP-net) is a graph model <*V*,* A*,* T*>, in which

(i) a set of variables makes up the** vertices** in the network,

(ii) a set of directed** arcs** connects pairs of vertices,

(iii) each vertex has a conditional preference** table** that qualifies the effects the parents have on the vertex.

The CP-net may be directed acyclic or directed cyclic graph [13]; i.e., it may exist with directed cycles, which is distinguished with classic Bayesian network [14, 15].

**Example 2 (auto configuration).**

For a car configuration, we focus on two attributes which are* Cl* (Class) and* Co*(Color), where* Cl* has no parents and* Co*'s parent is* Cl*. Assume the following conditional preferences:

(1) ClC≻ClBClC:  CoB≻CoCClB:  CoC≻CoB

The table is sufficient to order all the outcomes completely:

(2) $$\begin{array}{c} Cl_{C}Co_{B}≻Cl_{C}Co_{C}≻Cl_{B}Co_{C}≻Cl_{B}Co_{B} \end{array}$$

**Figure 1:**
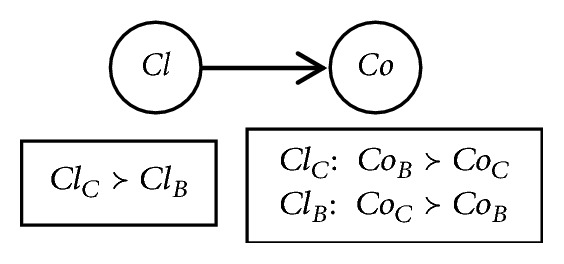
A CP-net of auto configuration.

The example can be described by the CP-net in Figure 1.

What calls for special attention is that we can get the preferred outcome on the basis of the conditional attributes it violates. The *Cl*_*C*_*Co*_*B*_ outcome violates none of the preference constraints. The *Cl*_*C*_*Co*_*C*_ outcome violates the conditional preference of* Co*. The *Cl*_*B*_*Co*_*C*_ outcome infringes the preference of* Cl*. The *Cl*_*B*_*Co*_*B*_ outcome violates both. From the above, we know the semantics of CP-nets implies that violating one child attribute has higher priority than violating the parent attributes [16].

**Definition 3 (dominant).**

In the decision space of a CP-net,* o*_*i*_⪰  *o*_*j*_ denotes that outcome* o*_*i*_ is equally dominant or more dominant compared with* o*_*j*_; if* o*  ⪰  *o*_*j*_ and* o*_*j*_  ⪰  *o*_*i*_, we say* o*_*i*_≻  *o*_*j*_ to denote that outcome* o*_*i*_ is strictly more dominant than* o*_*j*_[1].

Another preference order relation is indifferent to* o*_*i*_ and* o*_*j*_ if* o*_*i*_⪰  *o*_*j*_ and* o*_*j*_⪰  *o*_*i*_.

In this paper, we take strictly dominant relation as dominant because no weakly dominant relation is considered.

**Definition 4 (consistency).**

For CP-net* N*,* o*_*i*_ is an arbitrary outcome in the outcome space* Ω*, if no cycle sequence exists; that is, for some outcome, it does not appear more than one time in the arbitrary sequence; it is called consistent. For example, if there exists a sequence like* o*_1_≻  *o*_*i*_>≻  *o*_*j*_≻  *o*_1_, this CP-net does not satisfy consistency.

The consistency of CP-nets is closely related to the structure. Any CP-net with acyclic structure is consistent but not always as the cyclic CP-nets [8].

**Figure 2:**
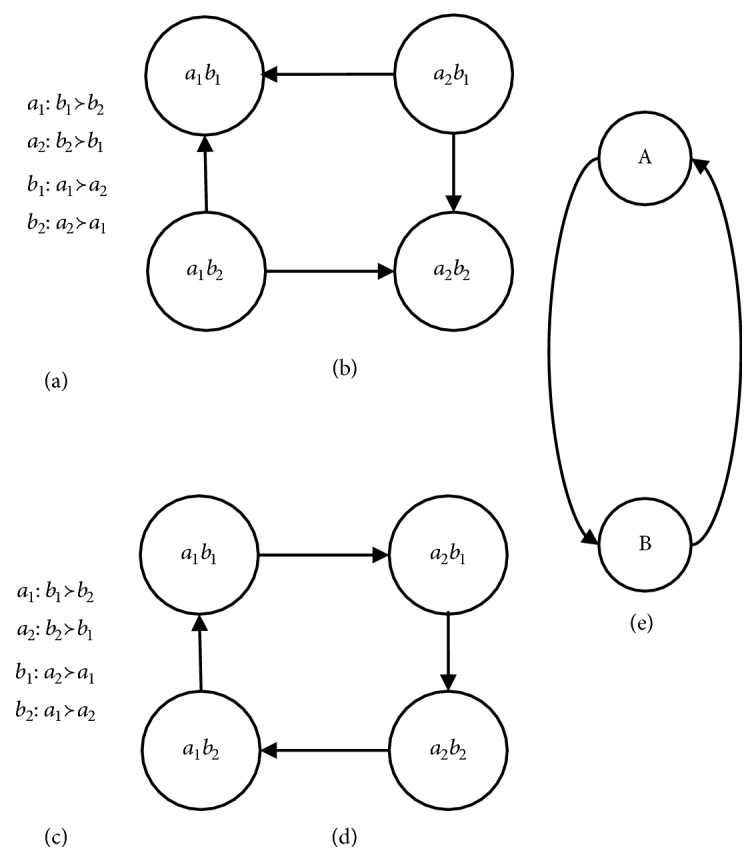
Examples of consistent and inconsistent cyclic CP-net over binary variables.

We distinguish between consistent and inconsistent cycle CP-nets by examples.  Figure 2 shows examples of consistent and inconsistent cyclic CP-net over binary variables. If the CPTs for this network are specified as in Figure 2(a), then the CP-net is consistent. However, if the CPTs are specified as in Figure 2(c), then the CP-net is inconsistent.

This paper deals with the problem of inconsistency in the formation of cyclic CP-nets and transforms the inconsistency problem into the consistency problem.

**Definition 5 (satisfiability).**

For a CP-net* N*, if there exists a preference ordering that satisfies all ceteris paribus preference assertions imposed by the CPTs, it is called satisfiability.

**Figure 3:**
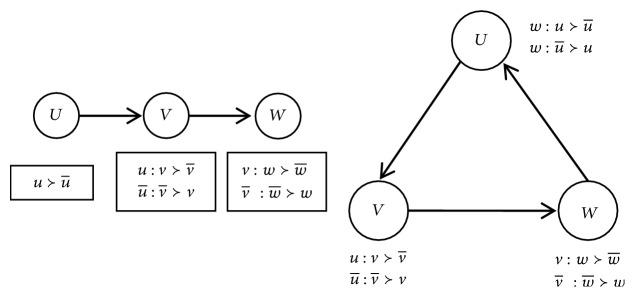
An acyclic CP-net and a cyclic CP-net with three same attributes.

But, acyclicity is not ensured by the semantics of CP-nets model, and acyclicity of the network automatically confers several important properties just like being satisfiable and consistency, while for cyclic CP-nets the situation is much more sophisticated. For example, for a binary-valued CP-net with three attributes, an acyclic one is showed on the left and the cyclic one is showed on the right in Figure 3. The cyclic CP-net is not inconsistent with omitted complicated reason. It also shows that the consistency of cyclic CP-nets is not guaranteed [2, 8].

We introduce the treewidth in graph theory to definite CP-nets with simple cycles. Treewidth is commonly used as a parameter in the complexity analysis of graph algorithms; many well-studied graph problems also have bounded treewidth.

**Definition 6 (tree decomposition).**

Given a graph* G* = (*V*,* E*), a tree decomposition is a pair (*X*,* T*), where* X=*{*X*_1_, ..., *X*_*n*_} is a set of subsets of* V*, and* T* is a tree whose nodes are the subsets* X*_*i*_, satisfying the following properties:

(1) Each graph vertex is associated with at least one tree node.

(2) For two vertices of the connection in the graph, there is a subset* X*_*i*_ that contains them.

(3) If* X*_1_ and* X*_2_ both contain a vertex* v*, then all nodes* X*_3_ of the tree in the path between* X*_1_ and* X*_2_ contain* v* as well.

**Definition 7 (treewidth).**

The treewidth is the size of its largest set* X*_*i*_ minus one, that is, max_*X*_*i*_∈*V*_⁡|*X*_*i*_| − 1. The treewidth of a graph* G* is the minimum width of tree decomposition of* G*.

The treewidth tw(*G*) of a graph* G* is the minimum width among all possible tree decomposition of* G*. The width of tree decomposition is the size of its largest set minus one.

**Definition 8 (CP-nets with simple cycles).**

A CP-net can be tree decomposed with treewidth bounded [14, 15] by constant* k*; it is a CP-net with simple cycles.

If a CP-net is a tree structure, the parent of a vertex is the vertex connected to it on the path to the root; every vertex except the root has a unique parent. A child of a vertex* v* is a vertex of which* v* is the parent.

But in the cyclic graph the parent is trouble. We take CP-nets with simple cycles into consideration in this paper. Several conditions need be considered.

**Definition 9 (parent of vertex in a cycle).**

When a vertex satisfies the following conditions, it is called a parent vertex in a cycle.

(1) The vertex does not connect with its lower level vertices apart from the cycle.

(2) The vertex connects with its high level vertices directly apart from the cycle.

(3) If the vertex satisfies (1) or (2), an arbitrary parent vertex is chosen.

**Figure 4:**
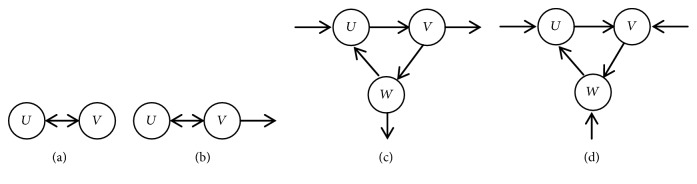
An acyclic CP-net and a cyclic CP-net with three same attributes.

In Figure 4, (a), (b), (c), and (d) are four types of parent.* U* or* V* is a random parent in (a);* U* is parent of* V* in (b);* U* is parent of* V* in (c);* U* or* V* or* W* is an arbitrary parent in (d).

From the reason of CP-nets of Boutilier [1], the following property is given.

**Property 10 .**

Parents have a larger positive impact on preference than that which children have.

That is, the nodes will have more impact when they are at higher level in a network, although we cannot compare two (or more) lower level impacts to impacts of one single parent constraint.

Algorithm 1 is based on the important property. FAS and FVS are presented in the following with cutting the cycles of CP-nets [15, 16].

## 4. Cutting Cycles by FAS

In this section, breadth-first search and depth-first search (DFS) are compared together and an algorithm of improved breadth-first search is presented.

**Definition 11 (FAS).**

For a CP-net* N*=<*V*,* A*,* T*> and a positive integer* k*, there exists a subset *X*⊆*V*with |*X*| ≤ *k* such that* N* with the arcs from* X* deleted is cycle-free [13].

In an undirected graph, FAS problem can be transformed into spanning tree of the graph and the arcs out of tree form the FAS. The time complexity of FAS is* O*(*nlogn*) because it is same as the spanning tree [10, 13].

In a directed graph, FAS is reduced to minimum capacity multicut problem in circular networks and its time complexity is *O*(*log*2|*X*|) [13, 14].

In the following, two search ways of depth-first search and breadth-first search in the graph theory are applied in FAS.

Depth-first search (DFS) traverses the tree vertices along the depth of the tree, as far as the search tree branch. When all of the vertices have already been explored, the search will go back to find the starting vertex. Then choose an arbitrary undiscovered vertex as a source vertex and repeat the above process; the whole process will be redone until all vertices have been visited so far [15, 16].

Breadth-first search (BFS) [17, 18] is limited to essentially two operations: (a) visit and check a vertex of a graph; (b) gain access to visit the vertices that neighbor the currently visited vertex. The BFS starts from the root vertex and checks all the neighboring vertices. Then for each of those neighbor vertices in turn, it checks their neighbor vertices which are unvisited, and so on. If the vertices are ergodic, the process is completed.

**Theorem 12 .**

The BFS is more reasonable than the DFS in breaking the cycles of CP-nets [19, 20].

**Proof**

From the properties and definition, the parent preferences have higher priority than the child preferences. The DFS starts from one parent to the children and then from another to the other children [21]. The BFS starts from one parent to another until every parent is visited and then visits the children. From the above process, the deleted vertices by BFS were children and the ones by DFS may be parents. So the BFS is more reasonable than the DFS.

For the length of all arcs are equal in CP-nets [22, 23], breadth-first search algorithm is the optimal solution, that is, the first solution it finds from the root of a certain minimum number of arcs; but for general figure [24] BFS does not necessarily return optimal solution. But this does not fit in the case of this paper. In this paper, an Improved BFS Algorithm of CP-nets is presented as in Algorithm 1.

**Example 13 .**

An example of cutting cycles is given by Algorithm 1.

**Figure 5:**
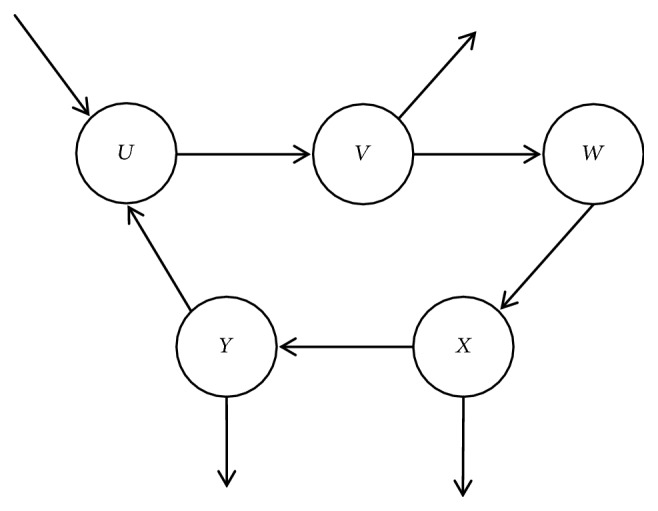
A cycle CP-net of 5 attributes in Example 13.

In the cycle CP-nets, the vertex does not connect with its lower level vertices apart from the cycle, or the vertex connects with its high level vertices directly apart from the cycle; we can use it as the vertex of a candidate's parents. That is, for any node* S*∈D, satisfy the following conditions: (1)  ∀ Son (S)∈D, (2) ∃ parent (S)∉D, so* S* can be the vertex of candidate parents. In Figure 5, for the node* U*, ∀ Son (*U*)∈D, ∃ parent (*U*)∉D. Add the nodes* U* to the candidate queue Q; for the node* V*, ∀ Son (*V*)∉D, ∃ parent (*V*)∈D. The nodes* V* do not add to the candidate queue. And we can finally get candidate queue Q = {*U*, *W*}. Because of size of (Q)>1, Rand() function is used to randomly select a node in Q to get nodes* U*. So the node* U* is the parents vertex of the cycle. And the edges between* U* and* Y* should be removed. Finally, cutting the cycle is successful.

**Theorem 14 .**

Improved BFS Algorithm of CP-nets is a sufficient condition for cutting the cycles of CP-nets.

**Proof**

Breadth-first search algorithm is complete. This means that, regardless of the type of graphics, as long as the target is present, it will be found by BFS.

## 5. Cutting Cycles by FVS

**Definition 15 (FVS).**

For a CP-net* N*=<*V*,* A*,* T*> and a positive integer* k*, there exists a subset *X*⊆*V*with |*X*| ≤ *k* such that* N* with the vertices from* X* deleted is cycle-free.

For general directed graphs, even with a method to convert FAS and FVS mutually in polynomial timing complexity put forward, the complexity of FVS is same as FAS [13].

The graph *G*[*V* − *X*] that remains after removing* X* from* G* is an induced forest. Thus, finding a minimum feedback vertex set in a graph is equivalent to finding a maximum induced forest. For an induced directed acyclic graph in the case of directed graphs, it is equivalent to finding the maximum one.

**Definition 16 (weighted vertex of a CP-net).**

Given a CP-net* N*= <*V, A, T*> where each vertex associates weight (*V*)=*c*, the minimum FVS problem is to find a min-weigh vertex.

From Definition 5, we know that parent vertex is more weighted than the child one. So the main work is to find and delete a less weighted vertex in a cycle.

In traditional attributes reduction, if we compare* o*_1_=* a*_1_*b*_1_*c*_1_*d*_1_*e*_1_ and* o*_2_=*a*_2_*b*_2_*c*_2_*d*_2_e_2_, we can compare* a*_1_*b*_1_*c*_1_*d*_1_ and* a*_2_*b*_2_*c*_2_*d*_2_ if* E* is decided by* D*. But we do not compare them because* E* is dependent only but not decided by* D*, and we may compare* a*_1_*b*_1_*c*_1_*e*_1_ and* a*_2_*b*_2_*c*_2_e_2_ if* D *is less weighted.

In the

**Property 17 (a deleted vertex).**

A vertex in a cycle is deleted when it satisfies the following conditions in the Figure 4:

(1) The weight of the vertex is minimal in the cycle.

(2) The vertex does not connect with other vertices out of the cycle because of* CPT*.

(3) When (1) and (2) have met together, (2) is considered at first.

In Figure 4, (a), (b), (c), and (d) are four types of deleted vertex.* U* or* V* is an arbitrary deleted vertex in (a);* U* is deleted because (2) of Property 17 in (b);* V* or* W* is an arbitrary deleted vertex in (c);* U*,* V, *or* W* is an arbitrary deleted vertex in (d).

See Algorithm 2.

**Example 18 .**

It is an example of cutting cycles by Algorithm 2.

**Figure 6:**
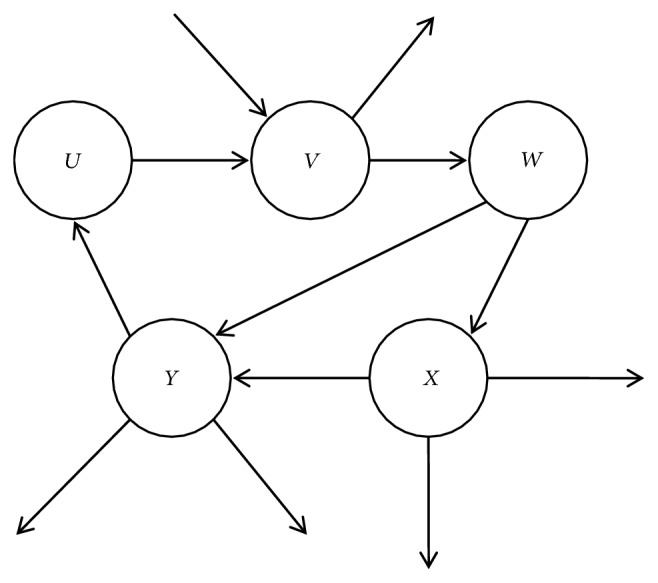
A cycle CP-net of 5 attributes in Example 18.

As shown in Figure 6, according to weight(*S*)=numSon(*S*)-numParent(*S*) in Algorithm 2, the weights of each node can be obtained as follows: weight(*U*)=0, weight(*V*)=0, weight(*W*)=-1, weight(*X*)=1, and weight(*Y*)=-1. The minimum value of weight is obtained by the min() function. In this example,* minWeight*=-1. All nodes satisfying the weight equal to -1 are added to P, getting P={*W*, *Y*}. According to Property 17, first delete vertices that are not connected to other vertices outside the cycle. In P, the node* W* is not connected with the outer node, so the node* W* is deleted.

Based on Algorithms 1 and 2, we give experiment to show the result of cutting cycles.

## 6. Experiments and Discussions

In order to evaluate the ability of our algorithms to deal with large volumes of data, we performed a set of experimental tests on synthetic data. We apply the random CP-nets generation algorithm in [25] to generate the CP-nets of the specified number of attributes. In this process, because our article is based on the structure of CP-nets, we only generate CP-nets structure without generating CPT. The experiment was carried out by the CP-nets of different structures. Our algorithms were implemented in Matlab and all the experiments were performed on a Windows 7 machine with 3.40 GHz clocked processor and 12 GB RAM.

**Figure 7:**
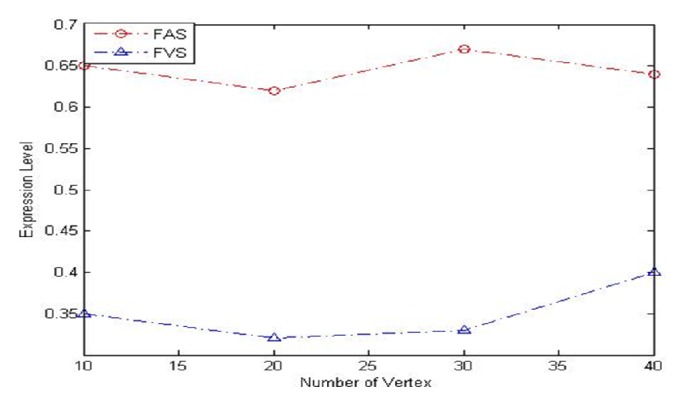
The expression of the CP-net after FAS and FVS.

Figure 7 shows expression of CP-nets [26, 27]. We can conclude that the FAS have more strong expressive capability.

**Figure 8:**
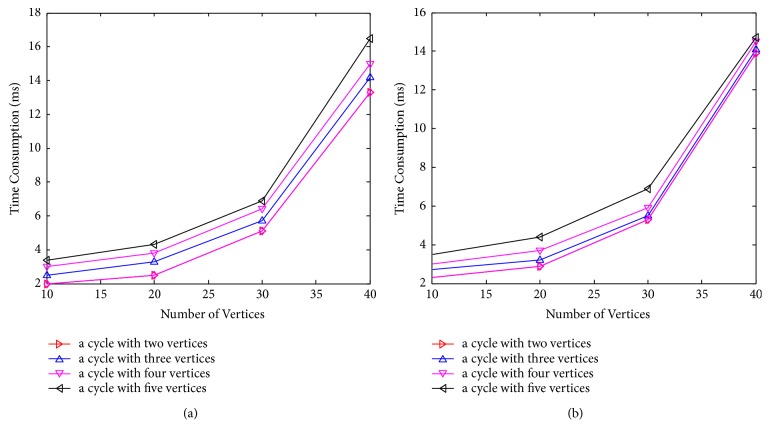
Time consumption of the FAS (a) and FVS (b) of one cycle.

**Figure 9:**
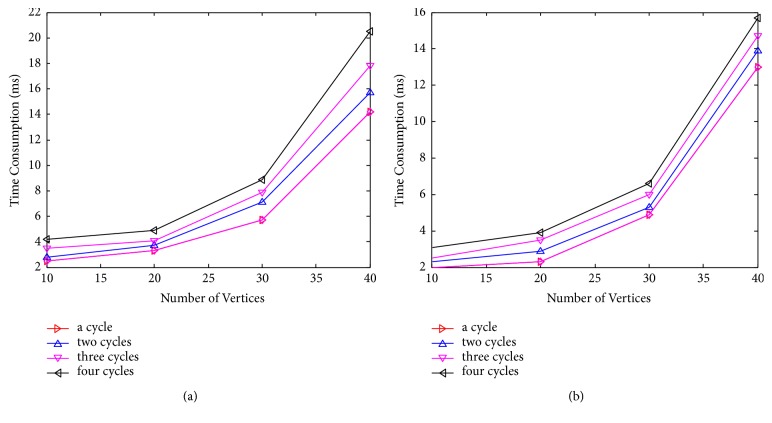
Time consumption of the FAS (a) and FVS (b) of different cycle.

Figure 8 shows time consumption comparison of FAS to FVS, which contains only one cycle and different number of vertices in the cycle [28, 29].  Figure 9 shows time consumption comparison of FAS to FVS, in which each cycle has three vertices and the number of cycles is different.

## 7. Conclusions and Future Work

Based on the theory of feedback vertex set and feedback arc set, this paper proposes a solution to the problem of cycles in CP-nets [30]. In particular, the definition of the parent vertices in the cycles of CP-nets greatly reduces the time complexity. Experiments show that the running time of the two methods is indifferent, but the expression ability is greatly different, and the feedback vertex set method has a great destruction to the completeness of CP-nets which is consistent with the theory proposed in the article. For the sake of simplification, we have not discussed the case of multiple values. But the cutting cycles problem of multivalued CP-nets also focuses on structure, not CPT. So this method is applicable to multivalued CP-nets, but we have not discussed this in depth in this paper; we will further study this issue in future research. Except for that, the use of heuristic methods [31, 32] to deal with cycles in a larger scale CP-net is very attractive.

## Figures and Tables

**Algorithm 1:**
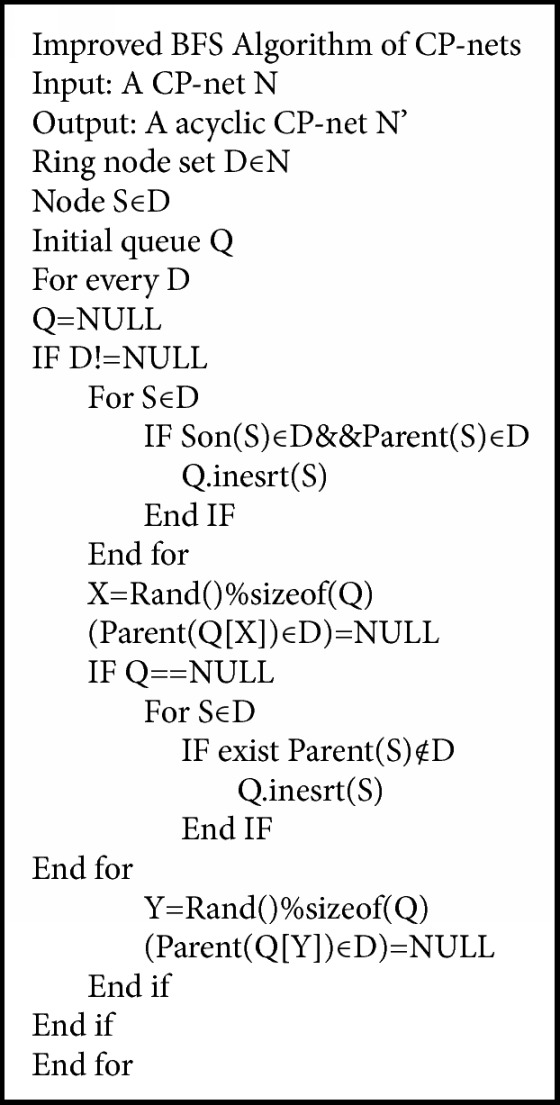
Improved BFS Algorithm of CP-nets.

**Algorithm 2:**
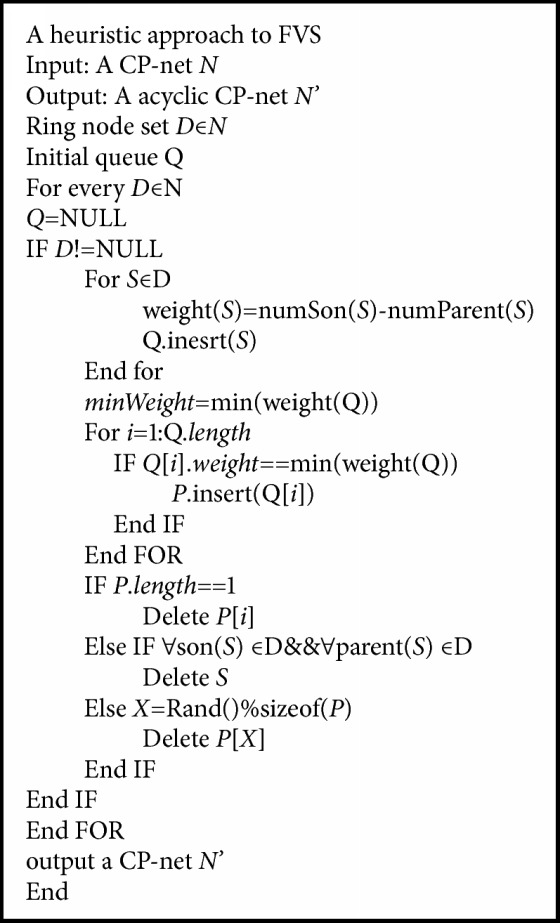
A heuristic approach to FVS.

## Data Availability

The data used to support the findings of this study are available from the corresponding author upon request.
